# Isolation and Characterization of a Thermotolerant Acetic Acid Bacteria Strain for Improved Zhenjiang Aromatic Vinegar Production

**DOI:** 10.3390/foods14050719

**Published:** 2025-02-20

**Authors:** Yuqin Wang, Shengkai Hua, Leyi Wang, Chunjia Bao, Xinnuo Chen, Xiang Wei, Yongjian Yu

**Affiliations:** School of Grain Science and Technology, Jiangsu University of Science and Technology, Zhenjiang 212100, China; yqwang@just.edu.cn (Y.W.); hsk1234562023@163.com (S.H.); 13238043020@163.com (L.W.); bcj1352374464@163.com (C.B.); 19741723953@163.com (X.C.); weixiang.xu@fomail.com (X.W.)

**Keywords:** Zhenjiang aromatic vinegar, solid-state fermentation, thermotolerant strain, *Acetobacter pasteurianus* TCBRC 103, aromatic compounds

## Abstract

This study aimed to isolate a thermotolerant acetic acid bacteria (AAB) strain from Zhenjiang aromatic vinegar (ZAV) and evaluate its potential as a starter culture for high-temperature solid-state vinegar fermentation. *Acetobacter pasteurianus* TCBRC 103 was successfully isolated and demonstrated superior thermotolerance compared to the industrial strain *A. pasteurianus* Huniang 1.01. *A. pasteurianus* TCBRC 103 exhibited robust growth and acetic acid production at 42 °C. When employed in the solid-state fermentation of ZAV, inoculation with *A. pasteurianus* TCBRC 103 resulted in higher fermentation temperatures, leading to enhanced accumulation of organic acids and volatile compounds. Notably, the concentrations of flavor compounds such as isoamyl acetate, acetic acid 2-phenylethyl ester, and acetoin were significantly higher in vinegar fermented by *A. pasteurianus* TCBRC 103 compared to those fermented by *A. pasteurianus* Huniang 1.01. Orthogonal partial least squares-discriminant analysis (OPLS-DA) identified 14 discriminative flavor compounds that could serve as potential markers for distinguishing between vinegars fermented by *A. pasteurianus* TCBRC 103 and *A. pasteurianus* Huniang 1.01. These findings highlight the promising application of *A. pasteurianus* TCBRC 103 as a starter culture for the production of high-quality ZAV under high-temperature conditions, with implications for reducing cooling costs and improving vinegar productivity in industry.

## 1. Introduction

Vinegars are widely used as acid condiments to improve the taste of cooked dishes. Furthermore, vinegars, especially the traditional ones in China, have the functions of controlling atherosclerosis, regulating lipid metabolism, decreasing high blood pressure, preventing heart disease, and relieving fatigue, and also have good therapeutic effects on headaches, diabetes, and liver injury [1,2,3,4]. Zhenjiang aromatic vinegar (ZAV) is one of the most representative traditional Chinese vinegars, and is famous for its rich fragrance and unique production technique [5,6,7]. ZAV is produced from grains (e.g., glutinous rice, barley, wheat bran, and rice hulls) during three main steps: alcohol fermentation, acetic acid fermentation (AAF), and an aging process [8]. AAF is considered the most critical phase [9]. The AAF stage employs wine, wheat bran, and rice hulls as substrates, utilizing a seed *Pei* to start solid-state fermentation of vinegar. During AAF, continuous vinegar *Pei* turning is used to prevent temperature accumulation, which could otherwise hinder microbial proliferation, while simultaneously supplying oxygen to aerobic bacteria such as AAB [10]. The vinegar *Pei*, primarily composed of wheat bran and rice hulls, contains abundant carbohydrates, vitamins, minerals, and proteins—essential nutrients for microbial growth. The vinegar *Pei*’ s porous structure allows air to freely circulate through ventilation ports, ensuring adequate oxygen supply for AAB to produce acetic acid [11]. During the AAF stage, *Acetobacter* spp. and *lactobacilli* spp. constitute the predominant microbial populations. *Acetobacter* spp. primarily synthesizes acetic acid, while *lactobacilli* spp. generates lactic acid. These bacterial strains exhibit strain-specific variations across different vinegar products. In ZAV, *A. pasteurianus* emerges as the dominant *Acetobacter* spp., with *L. acetotolerans* representing the primary *lactobacilli* spp. [12,13]. Moreover, other microorganisms including yeasts, bacillus, and fungi contribute to the fermentation of ZAV. This microbial diversity facilitates the synthesis of functional compounds such as ethyl acetate and tetramethylpyrazine [14,15,16,17].

Solid-state fermentation exhibits limited cooling efficiency, resulting in heat accumulation that typically elevates temperatures above 40 °C. A high fermentation temperature can accelerate the decomposition of amino acids and promote a reaction between the sugar, protein and amino acids, contributing to the production of high-quality vinegar characterized by a soft, rounded mouthfeel and rich aroma. However, most AAB cannot grow at temperatures exceeding 34 °C, presenting a critical limiting factor in vinegar production [9,18]. High temperature severely impacts some cellular components, such as massive protein misfolding and aggregation, and cell membrane damage [19,20]. To maintain optimal temperature conditions for AAB growth, a *Pei* turning machine, serving as a cooling system, is employed during the AAF process. However, this cooling system often operates inefficiently in high-temperature summer environments, leading to AAB mortality and substantial economic losses for enterprises. Therefore, the isolation of thermotolerant AAB species is essential for vinegar production to reduce cooling costs and enhance vinegar productivity.

AAB that can grow at 37 °C are considered thermotolerant strains [18]. Research over recent decades has identified several AAB capable of growth and acetic acid production at temperatures exceeding 40 °C [9,21,22,23,24,25,26,27]. For instance, *A. pasteurianus* TH-3, an adapted strain of *A. pasteurianus* SKU1108, which could grow at 41 °C and produced 3% acetic acid [23]. In addition, *A. pasteurianus* strains AF01 and CV01 exhibited robust growth at 41 °C, and produced acetic acid concentrations of 7.64% and 10.08%, respectively [25]. The most heat-resistant strain discovered to date is *A. pasteurianus* AAB4, isolated from Chinese vinegar *Pei*. This strain maintained alcohol-to-acid conversion rates of 77.1%, 14.5%, and 2.9% at 42 °C, 43 °C, and 44 °C, respectively [9]. 

The flavor profile of ZAV is primarily characterized by organic acids, amino acids, and volatile compounds [8]. The organic acids includes acetic acid, lactic acid, succinic acid, tartaric acid, malic acid, and citric acid, with acetic acid being predominant in ZAV, followed by lactic acid [12,28]. Acetic acid contributes the primary sour taste, and lactic acid serves to moderate the sharp sensory stimulation of acetic acid [4]. The amino acid profile of ZAV comprises 17–18 different amino acids, predominantly glutamic acid, tyrosine, serine, alanine, proline, and arginine [8,12]. These amino acids contribute distinct taste characteristics: glutamic acid provides the primary umami component and characteristic savory depth, tyrosine contributes bitterness, and serine adds sweetness. Together, these amino acids create a complex flavor matrix characteristic of ZAV [6]. Volatile compound analysis has identified between 33 and 88 different components in ZAV, primarily consisting of alcohols, acids, esters, ketones, and aldehydes [6,8,29,30]. Through the characterization of 22 ZAV samples, nine specific markers have been identified that distinguish ZAV from other Chinese vinegars: butylacetate, butanoic acid, 2-methylbutyric acid, 2-butanol, 3-methyl-1-butanol, 3-ethoxy-1-propanol, 2,5-dimethyl-hexanol, 2-acetyl furan, and 2,2-dimethyl-1,3-dioxolane-4-carboxaldehyde [30].

ZAV was inoculated by traditional seed *Pei*. After decades of long-term evolution, many strains in vinegar *Pei* have developed excellent fermentation performance, such as thermotolerance, and ethanol and acetic acid tolerance. In this study, a thermotolerant AAB strain, identified as *A. pasteurianus* TCBRC 103, was isolated from vinegar *Pei* of ZAV. The thermotolerance ability of this strain was further tested compared with a widely used AAB in Chinese industries called *A. pasteurianus* Huniang 1.01 [31]. In addition, we analyzed the quality of ZAV produced by *A. pasteurianus* TCBRC 103 through traditional solid-state fermentation at high temperature.

## 2. Materials and Methods

### 2.1. Isolation of Thermotolerant AAB 

The vinegar *Pei* containing AAB strains was collected from the workshop of Zhenjiang Vinegar Factory (Zhenjiang, China), where vinegar is produced through traditional solid-state fermentation. The samples were collected at a depth of approximately 15 cm from the surface at the tenth day of AAF, where the temperature of vinegar *Pei* was 49.2 °C. For AAB enrichment, 1 g of vinegar *Pei* was inoculated into 50 mL YPGE medium (1% yeast extract, 2% peptone, 2% glucose, and 4% ethanol) and incubated at 30 °C with shaking at 180 rpm for 24 h. The enrichment culture was diluted with sterile water and spread onto GYEC agar plates (2% glucose, 1% yeast extract, 2% peptone, 1.5% calcium carbonate, 3% ethanol, and 1.5% agar). After incubation at 40 °C for 3 days, candidate strains showing clear zones on GYEC agar plates were isolated and purified using the plate streaking. The pure cultured strain was further spread onto the GYEC agar plates and incubated at 40 °C for 3 days. On GYEC agar plates, calcium carbonate creates an opaque white background. When strains grow and produce acetic acid at high temperature (40 °C), the acid dissolves the surrounding calcium carbonate, forming clear zones around the colonies. The ratio between the clear zone diameter and colony diameter (potency index) indicates the strain’s acid production capacity. A higher potency index indicates greater acid production capacity [32,33]. The thermotolerant AAB showed the biggest potency index was selected and tested for acetic acid-producing capacity under a high temperature condition. 

The genomic DNA of the isolated thermotolerant AAB was extracted using a Bacterial Genomic DNA Isolation Kit (Sangon Biotech, Shanghai, China). The 16S rDNA sequence was then amplified from the genomic DNA using primers 27F (5′-AGAGTTTGATCCTGGCTCAG-3′) and 1492R (5′-ACGGTTACCTTGTTACGACTT-3′). The purified amplicons 16S rDNA gene was sequenced by the Sanger method and analyzed using the Basic Local Alignment Search Tool (BLAST, V2.14.0) available at the National Center for Biotechnology Information (NCBI) website (http://www.ncbi.nlm.nih.gov/, accessed on 30 June 2024).

### 2.2. Determination of Growth Under High-Temperature Condition

The temperature tolerance of *A. pasteurianus* TCBRC 103 and *A. pasteurianus* Huniang 1.01 was determined by spot assay and liquid culture assay. Each strain was cultured in YPGE medium shaken at 180 rpm for 24 h at 30 °C. For spot assay, the precultures were diluted with distilled water to an OD_600_ of 1. Cells (2 μL) at a dilution of 10^−1^, 10^−2^, 10^−3^, 10^−4^, and 10^−5^ were spotted on GYEC agar plates and incubated at different temperatures. For liquid culture assay, cell cultures were inoculated into fresh YPGE medium at an optical density (OD_600_) of 0.1, using 4–5 mL of preculture. The cultures were then incubated at 30 °C, 37 °C, 40 °C, or 42 °C with shaking at 180 rpm. Cell optical density at 600 nm was measured using a spectrophotometer (Hitachi, Tokyo, Japan). Dry cell weight was calculated according to the formula: Y = 0.3714X − 0.0285 (R² = 0.9929), where X represents OD_600_ and Y represents cell dry weight (g/L). 

### 2.3. Detection of Acetic Acid Concentration

The concentration of acetic acid was determined by high-performance liquid chromatography (HPLC) (Agilent, Santa Clara, CA, USA). The samples were initially centrifuged at 10,000× *g* for 10 min, and the supernatant was collected. Subsequently, the supernatant was filtered through a 0.22 μm membrane filter. A 10 µL aliquot of the filtered supernatant was then injected into the HPLC system. The HPLC was equipped with a UV detector (Agilent, Santa Clara, CA, USA) set at 210 nm and a Waters Atlantis dC18 column (Agilent, Santa Clara, CA, USA) (250 mm × 4.5 mm, 5 µm) maintained at 30 °C. The mobile phase consisted of 20 mmol/L NaH_2_PO_4_, which was adjusted to pH 2.70 with H_3_PO_4_, and the flow rate was set at 0.8 mL/min. Acetic acid content was quantified using external calibration standards of known concentrations.

### 2.4. Solid-State Fermentation of Traditional Chinese Vinegar

A mixture of raw materials, including 25 kg alcohol mash, 9 kg wheat bran and 4.5 kg rice hulls, was added to an 80 L jar. The alcohol mash was fermented by fungous and yeast (Anqi, Yichang, China) from steamed glutinous rice and contained 9% (*v*/*v*) ethanol. *A. pasteurianus* TCBRC 103 was precultured in YPD medium (1% yeast extract, 2% peptone, 2% glucose) for 24 h used as seed. The prepared seed broth was transferred to the surface of raw materials with 5% inoculum and then covered with 2 cm thickness of chaff. The jar was placed in a room temperature environment for 7 days for AAF. The fermented culture (termed *Pei* in Chinese) was turned once per day. 

### 2.5. Physiochemical Properties Analysis

Vinegar *Pei* was collected from top to bottom at five points (four vertexes and the center of the pool) and mixed thoroughly. Approximately 20 g of mixed vinegar *Pei* was transferred to a 250 mL flask, and then 60 mL of distilled water added. The mixture was then agitated using a rotary shaker at 100 rpm for 2 h at room temperature. Subsequently, the mixture was centrifuged for 10 min at 10,000× *g*, and the supernatant was collected for pH determination. The pH measurements were performed using a pH meter (FE28, Mettler Toledo, Shanghai, China).

The total acid content was determined according to the Chinese standard [34] and calculated as acetic acid per 100 g dry weight of vinegar *Pei*. Briefly, 10 g of vinegar *Pei* was transferred into a 250-mL Erlenmeyer flask containing 90 mL of distilled water. After adding 2–4 drops of phenolphthalein indicator solution (10 g/L), the mixture was titrated with 0.1 mol/L NaOH solution until a faint pink color persisted for 30 s. The volume of sodium hydroxide solution consumed was recorded as V_1_. A parallel blank determination was conducted using an equivalent volume of carbon dioxide-free water under identical conditions, and the volume of sodium hydroxide solution consumed was recorded as V_2_. The total acid content (expressed as acetic acid) was calculated using the following equation:

$$Total\;acids\;content=\frac{c\times (V1-V2)\times F\times 60}{m}$$

where c is the concentration of the standardized NaOH solution (mol/L). V_1_ is the volume of the sodium hydroxide solution consumed in sample titration (mL). V_2_ is the volume of the sodium hydroxide solution consumed in blank titration (mL). F is the sample dilution factor. M is the sample mass (g).

The reducing sugar content was measured using the 3,5-dinitrosalicylic acid (DNS) method [12]. The concentrations of six organic acids (acetic, lactic, succinic, tartaric, malic, and citric) were determined by HPLC using the same method described in Section 2.3. Each organic acid was quantified by the calibration curve of the corresponding authentic organic acids [35]. The temperature of vinegar *Pei* during the AAF stages was monitored using a thermometer (LL061, Bainianwumo, Hengshui, China).

### 2.6. Volatile Compounds Analysis

Volatile compounds were analyzed by Head Space Solid-Phase Microextraction-Gas Chromatography-Mass Spectrometry (HS-SPME/GC-MS) (Agilent, Santa Clara, CA, USA). Approximately 5 g of mixed vinegar *Pei* was placed in a 20 mL headspace bottle, and 0.5 g of NaCl and 10 μL of an internal standard (octan-2-ol, 25 mg/L) were added and mixed. The headspace bottle was tightly capped and heated for 10 min at 50 °C, and then extracted for 40 min using a SPME fiber (50/30 μm DVB/CAR/PDMS extraction head) at 50 °C. Subsequently, the extraction fiber was injected into the GC (Agilent, Santa Clara, CA, USA) injection port at 250 °C for a 5 min resolution time and used for GC-MS analysis. Separation was achieved using a DB-Wax column (30 m × 0.25 mm, 0.25 µm) with helium as the carrier gas at a flow rate of 1 mL/min and no split flow. The injection temperature was 250 °C. The temperature program was as follows: initial temperature was 40 °C, kept for 5 min, increased to 120 °C at a rate of 5 °C/min, then increased to 240 °C at a rate of 10 °C /min, kept for 5 min. MS conditions: EI ionization source, energy 70 eV, scanning range 30–500 m/z. The volatile compounds were identified by comparing the mass spectra with NIST17.L library. A semi-quantitative method was used to estimate the concentration of volatile compounds based on the added amount of octan-2-ol [29].

### 2.7. Statistical Analysis

OriginPro 2019b (V9.6.5.169, OriginLab, Northampton, MA, USA) software was used for hierarchical clustering analysis. Orthogonal partial least squares-discriminant analysis (OPLS-DA) models were created using the SIMCA 18 software (V18.0.1, Umetrics, Umeå, Sweden). All experiments in this study were performed at least three times. The data were presented as means ± standard error (SD) and analyzed by Student’s t-test at the significance of *p* < 0.05.

## 3. Results and Discussion

### 3.1. Screening and Identification of Thermotolerant AAB Strains 

Temperature is one of the key factors affecting the metabolism of AAB in the solid-state fermentation of vinegar [9,35]. The optimum growth temperature of AAB is 28–30 °C, and most strains exhibited no growth at temperatures above 34 °C [18]. However, in the process of the solid-state fermentation of vinegar, the temperature is usually higher than 40 °C, especially in summer, the high-temperature environment seriously restricts the industrial production of vinegar. Therefore, the present study aims to select a strain of AAB that is resistant to high temperature. 

**Figure 1:**
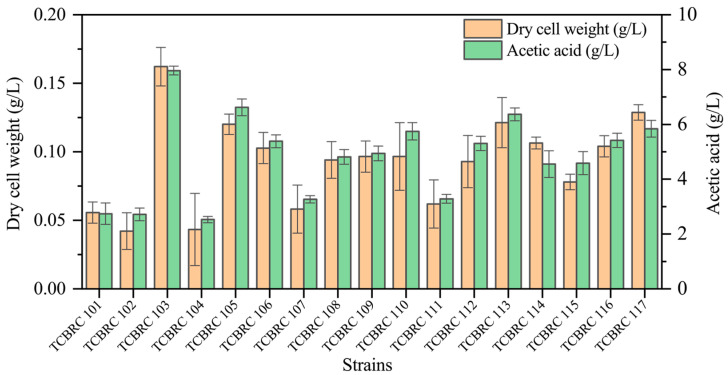
Screening of thermotolerant AAB strains that can produce acetic acid.

A total of 17 bacterial colonies showing a clear zone were observed on the GYEC medium at 40 °C in the initial screening experiment. These strains were sequentially tested for acetic acid-producing capacity under a high-temperature condition. After inoculation at 42 °C for 60 h, strain TCBRC 103 exhibited the highest cell growth and acetic acid-producing ability, which were0.16 ± 0.01 g/L dry cell weight and 7.96 ± 0.16 g/L acetic acid (Figure 1). Hence, strain TCBRC 103 was selected and utilized as the thermotolerant strain in the following study. The 16S rRNA gene amplicon was 1.5 kbp in size. Based on nucleotide-nucleotide BLAST analysis, strain TCBRC 103 was identified as *A. pasteurianus*, as it clustered with *A. pasteurianus* SX461 with 100% sequence similarity. 

### 3.2. Thermotolerance and Acetic Acid Yield of A. pasteurianus TCBRC 103

**Figure 2:**
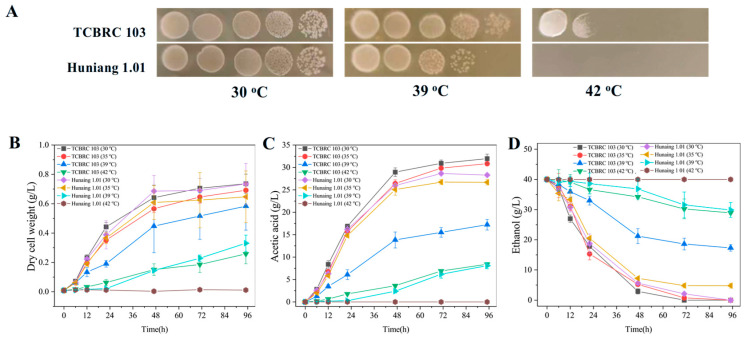
Temperature affects cell growth and acetic acid production in *A. pasteurianus* TCBRC 103 C. Comparing cell growth of *A. pasteurianus* TCBRC 103 and Huniang 1.01 on GYEC agar plates (**A**) or in liquid YPGE medium (**B**) under different temperature conditions. Comparing acetic acid production (**C**) and ethanol consumption (**D**) of *A. pasteurianus* TCBRC 103 and Huniang 1.01 under different temperature conditions.

*A. pasteurianus* Huniang 1.01, a widely utilized AAB strain in China industries [31], was used as the reference strain to evaluate the thermotolerance of *A. pasteurianus* TCBRC 103. Spot dilution assay showed that the cell biomass of the TCBRC 103 and Huniang 1.01 strains was similar at the optimal growth temperature of 30 °C. However, the cell biomass of the Huniang 1.01 strain was significantly inhibited at 39 °C, while the growth of the TCBRC 103 strain remained unaffected at this temperature. Notably, TCBRC 103 colonies were still observed at 42 °C, while the growth of Huniang 1.01 was completely repressed. To determine the quantitative disparities in thermotolerance between TCBRC 103 and Huniang 1.01, both strains were cultivated in liquid YPDE medium under various temperature conditions. As shown in Figure 2B, the biomass of TCBRC 103 was similar to that of Huniang 1.01 at 30 °C. The cell growth of both strains was not affected at 35 °C. In comparison, at 39 °C, the dry cell weight of the TCBRC 103 strain was 0.58 ± 0.16 g/L, which was 70.10% higher than that of Huniang 1.01. The growth of Huniang 1.01 was completely repressed at 42 °C, whereas TCBRC 103 could grow in liquid YPDE medium; the biomass of TCBRC 103 was 37.37% that of the control at 30 °C. These results indicated that TCBRC 103 was more tolerant to high-temperature stress, and was also higher than that previously reported for thermotolerant *A. pasteurianus* MSU10, which did not grow at 41 °C with 4% ethanol [36]. Thermotolerant AAB strains can withstand high-temperature stress through their extracellular membrane system and molecular chaperones [37]. The greater thermotolerance of *A. pasteurianus* TBCRC 103 may be attributed to the abundant pellicle polysaccharides, which function as a biofilm-like barrier protecting intracellular components from thermal damage. Additionally, high-temperature environments can induce the generation of reactive oxygen species (ROS), which can damage cellular macromolecules including proteins and DNA, severely impacting cell growth [38,39]. In our unpublished work, *A. pasteurianus* TBCRC 103 demonstrated superior ROS-scavenging efficiency compared to *A. pasteurianus* Huniang 1.01 under high-temperature conditions. Transcriptomic analysis revealed significant upregulation of ROS-scavenging genes (superoxide dismutases, glutathione peroxidases, and thioredoxin reductases) at elevated temperatures, suggesting that *A. pasteurianus* TBCRC 103 may employ an efficient antioxidant system to mitigate thermal stress.

The ability of heat-tolerant AAB to produce high levels of acetic acid at high temperatures is one of the most important factors in vinegar production. TCBRC 103 exhibited a slightly higher acetic acid synthesis capacity than Huniang 1.01 at an optimal growth temperature of 30 °C, yielding 31.96 ± 1.01 g/L compared to 28.29 ± 0.33 g/L, respectively. When the temperature was increased to 39 °C, the acetic acid yield of the TCBRC 103 strain remained high, at 17.24 ± 1.20 g/L, which was 2.13 times greater than that of Huniang 1.01. At 42 °C, TCBRC 103 maintained a production of 8.41 ± 0.41 g/L acetic acid, while Huniang 1.01 was unable to synthesize acetic acid due to the loss of its cellular activity (Figure 2C). The primary substrate for the production of acetic acid is ethanol. The consumption pattern of ethanol corresponds to the synthesis trend of acetic acid (Figure 2D). These results suggest that TCBRC 103 has a promising potential for application in AAF at high temperature.

### 3.3. Physicochemical Factors of ZAV During AAF

**Figure 3:**
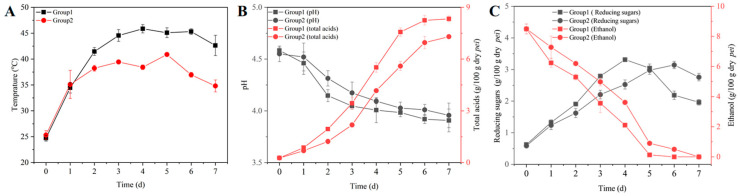
Dynamic changes in physicochemical parameters during fermentation of ZAV with *A. pasteurianus* TCBRC 103 and HuNiang 1.01. (**A**) Temperature; (**B**) total acids and pH; (**C**) reducing sugar and ethanol content. Group 1: ZAV fermented with *A. pasteurianus* TCBRC 103; Group 2: ZAV fermented with *A. pasteurianus* Huniang 1.01.

AAB are the key strains in the solid-state AAF of ZAV [40]. To investigate whether TCBRC 103 plays a positive role in AAF, we used Huniang 1.01 as a control and tested the physicochemical factors of ZAV fermented by TCBRC 103. The temperature, pH, total acids, and reducing sugar content in ZAV fermented by different groups (Group 1: TCBRC 103 and Group 2: Huniang 1.01) were measured at 0, 12, 24, 36, 48, 60, and 72 h. The temperatures of the two groups are shown in Figure 3A. During AAF, the temperature of both groups showed a trend of initially increasing and then decreasing. However, the temperature in Group 1 was higher than in Group 2, with Group 1 reaching temperatures above 40 °C on the second day of fermentation, which remained at a high temperature until the end of the fermentation process, peaking at 45.86 ± 0.81 °C. In contrast, the highest temperature in Group 2 was only 40.89 ± 0.36 °C. The likely reason is that TCBRC 103 exhibits greater heat tolerance compared to Huniang 1.01, enabling it to grow more effectively at high temperatures and generate more metabolic heat during AAF [41]. This increased heat production, in turn, leads to a higher fermentation temperature compared to that of Huniang 1.01. During AAF, the high fermentation temperature is essential for developing superior vinegar quality. High temperatures accelerate the decomposition of amino acids and promote reactions between sugars and proteins, as well as between sugars and amino acids, which enhances the accumulation of aromatic compounds [42]. However, higher fermentation temperatures can lead to the loss of volatile components. Notably, Es-sbata et al. [43] demonstrated that inoculation with AAB can mitigate the loss of volatile components. Furthermore, the utilization of thermotolerant AAB can significantly increase polyphenol content in vinegar, contributing to the production of high-quality vinegar.

The total acidity of ZAV is a crucial quality indicator [44]. During AAF, the pH in both groups exhibited a gradual decreasing trend, with Group 1 showing lower pH levels compared to Group 2 throughout the fermentation process (Figure 3A). Since pH is determined by acidic substances, we measured the changes in total acids content during fermentation. As shown in Figure 3B, the total acids in both fermentation systems continuously accumulated over time. At the ending stage of AAF (7 days), Group 1 exhibited a significantly higher acetic acid concentration (7.38 ± 0.26 g/100 g dry Pei) compared to Group 2 (6.36 ± 0.09 g/100 g dry Pei). 

Reducing sugars are important physicochemical indicators, which are produced through the microbial metabolism of raw materials and can be directly utilized by microorganisms to generate various flavor compounds [12]. As shown in Figure 3C, during the early stages of AAF, reducing sugars in both groups exhibited an increasing trend, primarily due to microorganisms enzymatically transforming starch from bran into reducing sugars. As fermentation progressed, the reducing sugars in Group 1 were consumed at a faster rate compared to Group 2. This could be attributed to the higher metabolic activity of TCBRC 103 in the high-temperature fermentation environment, which accelerated the metabolism and utilization of reducing sugars. These characteristics indicated that TCBRC 103 has superior qualities as a starter culture for AAF.

### 3.4. Organic Acids of ZAV During AAF

**Figure 4:**
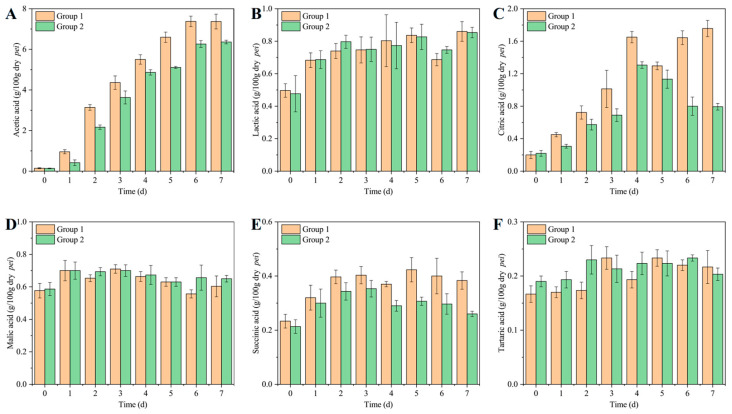
Dynamic changes in organic acids during fermentation of Zhenjiang aromatic vinegar with *A. pasteurianus* TCBRC 103 and Huniang 1.01. (**A**) Acetic acid; (**B**) lactic acid; (**C**) citric acid; (**D**) malic acid; (**E**) succinic acid, and (**F**) tartaric acid. Group 1: ZAV fermented with *A. pasteurianus* TCBRC 103; Group 2: ZAV fermented with *A. pasteurianus* Huniang 1.01.

Organic acids are key flavor components in vinegar [45]. Many of these acids also offer health benefits. In this study, HPLC was used to analyse six major organic acids—acetic, lactic, succinic, tartaric, malic, and citric—in ZAV. As shown in Figure 4, acetic acid is the most abundant acidic compound in ZAV. Acetic acid contributes to the vinegar’s strong pungency and short aftertaste [16]. The acetic acid concentration in Group 1 (7.38 ± 0.26 g/100 g dry *Pei*) was significantly higher than that in Group 2 (6.36 ± 0.09 g/100 g dry *Pei*), indicating that TCBRC 103 maintains a higher acetic acid synthesis capacity during solid-state vinegar fermentation (Figure 4A). The lactic acid levels showed no significant difference between the two groups. Since lactic acid is mainly produced by lactic acid bacteria, its synthesis can be influenced by interactions with *Acetobacter* spp. In this study, both TCBRC 103 and Huniang 1.01 had similar effects on lactic acid bacteria, leading to no noticeable variation in lactic acid content between the two groups (Figure 4B). Tartaric acid, malic acid, citric acid, and succinic acid are all non-volatile organic acids (Figure 4C–F). While tartaric and malic acid levels remained similar between the groups, Group 1 contained significantly higher levels of citric and succinic acids compared to Group 2. Succinic acid is an intermediate metabolite in the tricarboxylic acid cycle, and also serves as a precursor in other complex metabolic pathways, resulting in lower accumulation and yields during acetic acid fermentation [46]. Despite the small amount, succinic acid can reconcile the sharp taste of acetic acid and improve the sensory quality of ZAV. 

### 3.5. Flavor Profiling of ZAV During AAF

**Figure 5:**
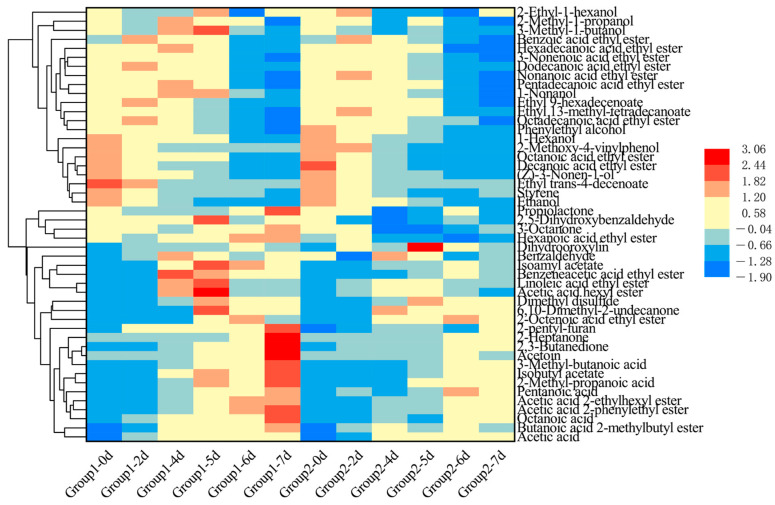
Heatmap of the flavor metabolites during fermentation of ZAV with *A. pasteurianus* TCBRC 103 and Huniang 1.01. Group 1: ZAV fermented with *A. pasteurianus* TCBRC 103; Group 2: ZAV fermented with *A. pasteurianus* Huniang 1.01.

The volatile flavor compounds in vinegar are the primary source of the rich aroma of ZAV. The presence of these volatile compounds contributes to a more balanced fragrance and enhances the complexity of the overall taste profile of the vinegar [29]. The volatile components of ZAV were determined semi-quantitatively using HS-SPME-GC-MS. A total of 48 volatile compounds were identified in ZAV during AAF with different strains, including 22 esters, 5 acids, 9 alcohols, 6 ketones, 2 aldehydes, and 4 others. The concentrations of volatile compounds were converted into a heat map (Figure 5). 

Acids were the most abundant volatile compounds in ZAV, and their concentration increased as fermentation progressed. At the end of fermentation, the volatile acid content in Group 1 was significantly higher than that in Group 2. These volatile acids included acetic acid, octanoic acid, pentanoic acid, 3-methyl-butanoic acid, and 2-methyl-propanoic acid. The odor descriptor of these volatile acids is shown in Table S1. Notably, the 2-methyl-propanoic acid content in Group 1 was 2.76 times higher than that in Group 2. 2-Methyl-propanoic acid is characterized by buttery, fatty, and sour aromas [47], and this compound was absent in the early stages of fermentation, with its concentration gradually increasing as fermentation progressed.

Esters were the most diverse category of volatile compounds. The ester content in Group 1 was significantly higher than that in Group 2, which may be attributed to the fact that esters are formed through the dehydration condensation of acids and alcohols; a reaction that is favored in high-temperature environments. Since the fermentation temperature in Group 1 was higher than that in Group 2, the elevated fermentation conditions promoted ester synthesis in Group 1. Among all the esters, isoamyl acetate had the highest concentration. In Group 1, after 5 days of fermentation, the concentration reached 8717.02 ± 48.42 g/100 g dry *Pei*; significantly higher than the 1548.75 ± 33.53 g/100 g dry *Pei* observed in Group 2. Isoamyl acetate is characterized by a pear or banana-like fruity aroma [48], contributing to the characteristic fruity scent of ZAV.

The content of acetic acid 2-phenylethyl ester and isobutyl acetate increased significantly during AAF. In Group 1, the concentration of acetic acid 2-phenylethyl ester rose from 46.63 ± 1.03 g/100 g dry *Pei* in the early fermentation stage to 2856.97 ± 27.7 g/100 g dry *Pei*, which was significantly higher than the final concentration in Group 2 (1556.58 ± 25.72 g/100 g dry *Pei*). Acetic acid 2-phenylethyl ester is synthesized through the dehydration condensation of acetic acid and phenylethyl alcohol. Phenylethyl alcohol is primarily synthesized by yeast strains during the alcoholic fermentation phase, and as acetic acid continues to be synthesized during AAF, the formation of acetic acid 2-phenylethyl ester is promoted, leading to a gradual increase in its concentration. This ester has a rose-like fragrance, contributing to the floral aroma of ZAV. Isobutyl acetate has a pineapple-like fragrance [49]; its concentration in Group 1 increased from 20.67 ± 0.46 g/100 g dry *Pei* in the early stage to 1657.42 ± 75.43 g/100 g dry *Pei*, significantly higher than the 821.19 ± 23.43 g/100 g dry *Pei* found in Group 2 at the end of fermentation. Compounds such as benzeneacetic acid ethyl ester, acetic acid 2-ethylhexyl ester, 2-octenoic acid ethyl ester, and linoleic acid ethyl ester were absent during the alcoholic fermentation stage but gradually accumulated during AAF. In Group 1, by the end of fermentation, their concentrations reached 156.28 ± 4.23 g/100 g dry *Pei*, 167.24 ± 5.48 g/100 g dry *Pei*, 29.76 ± 0.49 g/100 g dry *Pei*, and 103.27 ± 2.75 g/100 g dry *Pei*, respectively. The reason these compounds are only produced during AAF is that a large number of acidic compounds are synthesized during AAF, which then undergo dehydration condensation with alcohols to form the corresponding esters. Additionally, some esters, such as octanoic acid ethyl ester, nonanoic acid ethyl ester, dodecanoic acid ethyl ester, decanoic acid ethyl ester, and hexadecanoic acid ethyl ester, showed a gradual decrease in concentration during AAF. This reduction may be due to their instability and subsequent degradation by a microorganism.

The concentration of alcoholic compounds gradually decreases during AAF. Ethanol, the primary substrate, is converted to acetic acid by membrane-bound alcohol dehydrogenase (ADH) and aldehyde dehydrogenase (ALDH) enzymes in AAB. Phenylethyl alcohol, a major aromatic compound produced during alcohol fermentation, undergoes esterification with acetic acid to form acetic acid 2-phenylethyl ester, resulting in a significant reduction in its concentration during AAF.

The concentration of ketone compounds increases significantly as fermentation progresses, with acetoin showing the most prominent increase. Acetoin is characterized by a pleasant yogurt aroma and a fatty, creamy butter taste [50]. It is considered an important aroma-active compound and a precursor of bioactive molecules, with its biosynthesis in vinegars garnering considerable attention [14,51]. *A. pasteurianus* are the primary acetoin-producing strains. At the end of fermentation, the acetoin concentration in Group 1 (4595.92 ± 47.05 g/100 g dry *Pei*) was significantly higher than that in Group 2 (682.18 ± 21.18 g/100 g dry *Pei*), suggesting that TCBRC103 exhibits a stronger acetoin synthesis ability compared to Huniang 1.01. 2,3-Butanedione is associated with butter, sweet, and cream aromas [51]. The concentration of 2,3-butanediol gradually increases during AAF, rising from 4.22 ± 0.05 g dry *Pei* at the start of fermentation to 495.84 ± 16.77 g/100 g dry *Pei* in Group 1. In Group 2, at the end of fermentation, the concentration of 2,3-butanediol was 101.17 ± 2.94 g/100 g dry *Pei*.

**Figure 6:**
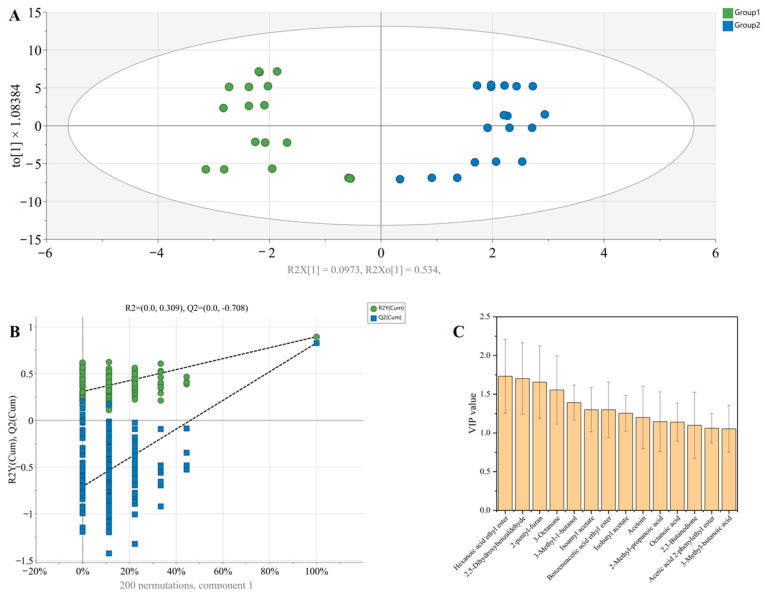
OPLS-DA analysis of volatile compounds of ZAV with *A. pasteurianus* TCBRC 103 and Huniang 1.01. (**A**) Score scatter plot; (**B**) permutation test; (**C**) VIP plot. Group 1: ZAV fermented with *A. pasteurianus* TCBRC 103; Group 2: ZAV fermented with *A. pasteurianus* Huniang 1.01.

The OPLS-DA model was applied to differentiate between Group 1 and Group 2 (Figure 6). The independent variable fitting index (R²x), the dependent variable fitting index (R²y), and the model prediction index (Q²) were 0.915, 0.896, and 0.829, respectively (Figure 6A). As both R² and Q² values exceeded 0.5, the model fitting was considered acceptable [52]. Permutation tests (*n* = 200) confirmed the validity of the original model (Figure 6B). These results suggest that the model verification was effective and that the findings could be reliably used for the identification and analysis of vinegar aroma.

Variable importance in projection (VIP) was utilized to quantify the contribution of each variable to the classification in the OPLS-DA model. In the present study, a total of 14 discriminative flavor compounds (VIP > 1 and *p* < 0.05) were identified as distinguishing factors between Group 1 and Group 2. These compounds included hexanoic acid ethyl ester, 2,5-dihydroxybenzaldehyde, 2-pentylfuran, 3-octanone, 3-methyl-1-butanol, isoamyl acetate, benzeneacetic acid ethyl ester, isobutyl acetate, acetoin, 2-methyl-propanoic acid, octanoic acid, 2,3-butanedione, acetic acid 2-phenylethyl ester, and 3-methyl-butanoic acid (Figure 6C). These differential volatile compounds may serve as potential biomarkers for differentiating between vinegars fermented by TCBRC 103 and those fermented by Huniang 1.01. 

## 4. Conclusions

In this study, a thermotolerant AAB, *A. pasteurianus* TCBRC 103, was successfully isolated from ZAV. Compared to the industrial strain *A. pasteurianus* Huniang 1.01, TCBRC 103 exhibited superior thermotolerance, with the ability to grow and produce acetic acid at temperatures up to 42 °C. When utilized as a starter culture for the solid-state fermentation of ZAV, TCBRC 103 demonstrated several advantageous characteristics. *A. pasteurianus* TCBRC 103 achieved higher fermentation temperatures, leading to increased accumulation of organic acids and volatile flavor compounds. Notably, TCBRC 103 produced significantly higher levels of key aromatic compounds such as isoamyl acetate, acetic acid 2-phenylethyl ester, and acetoin compared to Huniang 1.01. OPLS-DA analysis identified 14 discriminative flavor compounds that could potentially serve as markers for distinguishing between vinegars fermented by these two strains. These findings highlight the potential of *A. pasteurianus* TCBRC 103 as a superior starter culture for the production of high-quality ZAV, particularly under elevated temperature conditions. The application of this thermotolerant strain could help reduce cooling costs and improve vinegar productivity in industrial settings. 

## Data Availability

The original contributions presented in the study are included in the article/Supplementary Material; further inquiries can be directed to the corresponding author.

## Supplementary Materials

The following supporting information can be downloaded at: https://www.mdpi.com/article/10.3390/foods14050719/s1, Table S1: Odour descriptor of volatile aroma compounds in ZAV.

## Abbreviations

AAB	Acetic acid bacteria
ZAV	Zhenjiang aromatic vinegar
AAF	Acetic acid fermentation
ALDH	Aldehyde dehydrogenase
ADH	Alcohol dehydrogenase
