# Supplementary material for: Isolation and Characterization of a Thermotolerant Acetic Acid Bacteria Strain for Improved Zhenjiang Aromatic Vinegar Production
Source: Foods. 2025 Feb 20;14(5):719. doi: 10.3390/foods14050719 (PMC11898446; doi:10.3390/foods14050719)
Supplement: Supplementary file 1 [file foods-14-00719-s001.zip › foods-3421790-supplementary.pdf]

Article

Isolation and Characterization of a Thermotolerant Acetic Acid Bacteria Strain for Improved Zhenjiang Aromatic Vinegar Production

Yuqin Wang [1], Shengkai Hua [1], Leyi Wang [1], Chunjia Bao [1], Xinnuo Chen [1], Xiang Wei [1] and Yongjian Yu [1,*]

[1] School of Grain Science and Technology, Jiangsu University of Science and Technology, Zhenjiang 212100, China
* Correspondence: yuyj@just.edu.cn (Y.J. Yu)

Table S1 Odour descriptor of volatile aroma compounds in ZAV

Category	CAS	Compounds	Odour descriptor
Acids	64-19-7	Acetic acid	Sour, pungent
	124-07-2	Octanoic acid	Rancid, fatty
	110-54-3	Pentanoic acid	Unpleasant, sharp
	503-74-2	3-Methyl-butanoic acid	Sweaty, cheese-like
	79-31-2	2-Methyl-propanoic	buttery, fatty, and sour
Esters	123-92-2	Isoamyl acetate	pear or banana-like
	103-45-7	2-Phenylethyl ester	rose-like
	110-19-0	Isobutyl acetate	pineapple-like
	10031-42-0	Benzeneacetic acid ethyl ester	sweet, fruity
	103-09-3	Acetic acid 2-ethylhexyl ester	sweet, fruity
	87741-46-4	2-Octenoic acid ethyl ester	Mild, slightly fatty
	4130-42-9	Linoleic acid ethyl ester	fatty
	106-32-1	Octanoic acid ethyl ester	Fruity, apple-like
	123-29-5	Nonanoic acid ethyl ester	Sweet, fruity
	106-33-2	Dodecanoic acid ethyl ester	Mild, fatty, waxy
	110-42-9	Decanoic acid ethyl ester	Fruity, fatty
	628-97-7	Hexadecanoic acid ethyl ester	Mild, waxy
Alcohols	64-17-5	Ethanol	pirituous, wine-like
	60-12-8	Phenylethyl alcohol	rose-like, floral fragrance
Ketones	513-86-0	Acetoin	pleasant yogurt aroma
	431-03-8	2,3-Butanedione	butter, sweet, and cream aromas
